# Perceived HR practices and employee performance: mediating effects of affective commitment and moderating effects of transformational leadership

**DOI:** 10.3389/fsoc.2026.1840147

**Published:** 2026-06-17

**Authors:** Imane Hasinat, Malika Soulami, Salma Nassim, Saad Benchekroun, Sofia Loulidi

**Affiliations:** 1 LAREM Laboratory (Laboratory of Multidisciplinary Research), HECF Business School, Sidi Mohamed Ben Abdellah University, Fez, Morocco; 2 Laboratory Synergie-Lab, Polydisciplinary Faculty of Errachidia (FPE), Moulay Ismail University, Meknes, Morocco; 3 Laboratory REMMALIF, Higher School of Technology (EST), Sidi Mohamed Ben Abdellah University, Fez, Morocco

**Keywords:** affective commitment, employee performance, individual performance, perceived HRM practices, transformational leadership

## Abstract

**Orientation:**

In a context characterized by intense competition and profound organizational change, individual employee performance has become a strategic concern for organizations. Human resource management practices play a central role in shaping employee attitudes and behaviors, which highlights the need to better understand how these practices influence individual performance.

**Research purpose:**

This study investigates the impact of perceived human resource management (HRM) practices on multidimensional individual performance in Moroccan small and medium-sized enterprises (SMEs), examining the mediating role of affective commitment and the moderating effect of transformational leadership.

**Motivation for the study:**

Despite the growing body of research examining the relationship between human resource management practices and employee performance, limited studies have simultaneously integrated the mediating role of affective commitment and the moderating role of transformational leadership. In a context characterized by rapid organizational transformations, understanding these mechanisms is crucial for supporting sustainable organizational performance.

**Research approach and method:**

This study adopted a quantitative research design. Based on an extensive literature review, a conceptual model and research hypotheses were developed. Data were collected through an online questionnaire administered to Moroccan employees, yielding 152 valid responses, which were analyzed using partial least squares structural equation modeling (PLS-SEM).

**Main findings:**

The results show that perceived HR practices positively influence task, contextual and adaptive performance while reducing counterproductive work behavior. The findings also confirm the partial mediating role of affective commitment and the moderating role of transformational leadership in this relationship.

**Practical/managerial implications:**

The findings suggest that organizations should implement effective and supportive HR practices to enhance employee performance and strengthen affective commitment. In addition, fostering transformational leadership behaviors may reinforce the positive effects of HR practices on employee outcomes and contribute to improved organizational performance.

**Contribution/value add:**

This study contributes to the HRM–performance literature by proposing and empirically testing a moderated mediation model that integrates perceived HR practices, affective commitment and transformational leadership in explaining individual performance. By providing empirical evidence from the Moroccan context, the study extends existing research on the mechanisms through which HR practices influence employee outcomes in emerging economies.

## 1 Introduction

Global economic volatility, accelerated digital transformation, and the evolving nature of work arrangements have profoundly reshaped organizational priorities. In this context, enhancing individual performance while maintaining strong employee engagement has become a central concern for organizations (Schilirò, 2021; Kniffin et al., 2021). Consequently, human resource management (HRM) is increasingly recognized as a strategic lever capable of influencing employees' attitudes, behaviors, and organizational effectiveness (Bakker and Bal, 2010; Giancaspro et al., 2022).

However, the literature indicates that the relationship between HRM practices and employee performance cannot be understood as a direct, automatic, or linear link. Rather, it is shaped by complex social processes, implementation dynamics, and contextual contingencies (Bowen and Ostroff, 2004; Purcell and Kinnie, 2007; Boxall and Macky, 2009). Recent research further confirms this complexity by showing that HRM effects are conditioned by multiple mediating mechanisms and contextual factors, such as organizational culture, leadership, and employees' cognitive processes, thereby highlighting their fundamentally contingent and multi-determined nature (Yang and Mostafa, 2024).

Within this perspective, a growing body of literature emphasizes that the effectiveness of HRM largely depends on employees' perceptions and interpretations of organizational practices (Nishii et al., 2008a; Den Hartog et al., 2013; Wang et al., 2020). This shift from intended to perceived HR practices highlights the central role of employees' cognitive and interpretative processes in shaping work-related attitudes and behaviors. Nevertheless, despite these advances, the underlying mechanisms remain only partially understood, reflecting the persistence of the “black box problem” in HRM research (Jiang et al., 2012; Guest, 2017).

From the perspective of Social Exchange Theory (Blau, 1964; Cropanzano and Mitchell, 2005), employees tend to reciprocate favorable organizational treatment through positive attitudes and behaviors. When HRM practices are perceived as fair and supportive, they foster the development of affective commitment and encourage positive work behaviors. Affective commitment, defined as the emotional attachment to and identification with the organization (Fernández-Lores et al., 2016; Meyer and Allen, 1991), has consistently been associated with higher performance and lower counterproductive behaviors (Koopmans et al., 2011; Alfes et al., 2012; Mostafa et al., 2023). Moreover, empirical evidence confirms its mediating role in the relationship between HRM and performance (Jiang et al., 2012; Aktar and Pangil, 2018).

Despite these advances, three major theoretical tensions continue to limit an integrated understanding of the HRM–performance relationship. First, employee performance has historically been organizational as a unidimensional construct focused on task performance. However, foundational studies distinguish between task performance and contextual performance (Borman and Motowidlo, 1993; Motowidlo and Van Scotter, 1994), a distinction later extended to include adaptive performance and counterproductive work behaviors (Campbell, 1990; Koopmans et al., 2014; Saidin et al., 2024). Nevertheless, despite these conceptual developments, empirical studies continue to organizational performance in a fragmented and often partial manner. Recent research highlights substantial variability in measurement approaches, ranging from unidimensional indicators to non-standardized composite constructs (Ramawickrama et al., 2017; Hasinat et al., 2024; Reig-Botella et al., 2024). This heterogeneity limits comparability across studies and constrains cumulative knowledge development regarding HRM effects. This tension underscores the need for a genuinely multidimensional and integrated approach to employee performance (Aguinis et al., 2024).

Second, although extensive research has examined the relationship between HRM practices and performance, many studies continue to focus on direct effects, thereby neglecting the underlying psychological mechanisms. Recent literature strongly calls for opening the “black box” by incorporating mediating variables such as attitudes, perceptions, and commitment (Nishii et al., 2008b; Jiang et al., 2012; Guest, 2017). However, integrated models simultaneously combining perceived HRM practices, affective commitment, and multidimensional performance remain limited. This reflects a tendency to reduce complex psychological processes to generic attitudinal mediators, without fully capturing the cognitive and interpretative mechanisms through which employees make sense of organizational practices.

Third, recent research increasingly recognizes that HRM effectiveness is strongly shaped by contextual and relational factors. Leadership plays a central role in how HRM practices are interpreted, enacted, and translated into employee behaviors. Transformational leadership enhances employees' motivation, sense-making processes, and engagement (Crede et al., 2019; Nikolova et al., 2019), and amplifies the effects of HRM practices on employee outcomes (Hai et al., 2020). However, HRM systems and leadership are still largely examined as separate analytical streams, with limited attention to their joint effects. Few studies simultaneously integrate mediation and moderation mechanisms within a unified analytical framework, thereby limiting understanding of the complex interactions between organizational systems and managerial dynamics. This limitation calls for an integrated model combining mediation and moderation within a broader contingency perspective.

In response to these limitations, this study develops and empirically tests a moderated mediation model linking perceived HRM practices to multidimensional employee performance through affective commitment, while incorporating transformational leadership as a moderating variable. The analysis is based on PLS-SEM and data collected from employees in Morocco.

This research contributes to the HRM literature in three main ways. First, it adopts a multidimensional approach to employee performance, enabling a more comprehensive understanding of work-related behaviors. Second, it simultaneously integrates psychological mechanisms (affective commitment) and contextual mechanisms (transformational leadership) within a unified explanatory framework. Finally, by focusing on the Moroccan context, it provides empirical evidence from an emerging economy, thereby enhancing the external validity of HRM models.

## 2 Literature review

### 2.1 Perceived human resource practices and employee performance

The contemporary world of work has undergone profound transformations driven by globalization„ rapid technological advancements, and the increasing prevalence of remote and flexible work arrangements (Schilirò, 2021; Kniffin et al., 2021). These structural changes have reshaped organizational systems and intensified talent competition. Consequently, organizations are increasingly seeking effective strategies to enhance employee performance while maintaining high levels of employee commitment and retention (Sinisterra et al., 2024; Luna-Arocas and Danvila-del-Valle, 2024).

From a theoretical perspective, the employment relationship extends beyond formal contractual arrangements and is embedded in social exchange processes characterized by mutual obligations, trust, and perceived fairness between the organization and its employees. The Social Exchange Theory, originally developed by Homans (1958) and further elaborated by Blau (1964), posits that social relationships evolve through reciprocal exchanges of resources and benefits.

In behaviorionl contexts, employees evaluate the balance between the benefits they receive and the efforts they invest in their work relationships (Cropanzano and Mitchell, 2005). When employees perceive that the organization provides valuable resources, support, and fair treatment, they are more likely to reciprocate through positive attitudes and behaviors that contribute to organizational effectiveness.

Within this perspective, human resource management practices represent a key mechanism through which organizations influence employee outcomes. HR practices such as recruitment, training, performance appraisal, and compensation are designed to enhance employees' abilities, motivation, and opportunities to contribute effectively to organizational objectives (Bakker and Bal, 2010; Giancaspro et al., 2022). However, the effectiveness of these practices depends not only on their formal implementation but also on employees' perceptions and interpretations of them.

In addition to the social exchange perspective, the relationship between HR practices and performance can be explained through the Ability–Motivation–Opportunity (AMO) framework. According to this framework, initially formalized by Wright and McMahan (1992) and further developed by Appelbaum et al. (2000), HR practices enhance employee performance by strengthening three critical mechanisms: employees' abilities through training and skill development, their motivation through rewards and performance management systems, and their opportunities to contribute through participative and supportive work environments. Empirical studies have validated the explanatory power of the AMO framework in linking HR practices to employee performance outcomes (Jiang et al., 2012).

Employees' perceptions of HR practices constitute a critical mechanism linking organizational policies to individual behavioral outcomes. Individuals interpret HR practices based on their experiences, expectations, and personal values, leading to variations in how organizational initiatives are understood and evaluated (Khilji and Wang, 2007; Nishii et al., 2008a). Consequently, the impact of HR practices on employee behavior largely depends on employees' subjective perceptions of the support, fairness, and developmental opportunities embedded in these practices (Den Hartog et al., 2004; Alfes et al., 2012).

Empirical research indicates that when employees perceive that their organization provides both economic and socio-emotional support, they tend to reciprocate through favorable attitudes and behaviors that contribute to improved performance (Rhoades and Eisenberger, 2002; Bowra et al., 2012). Positive perceptions of HR practices, therefore, foster higher levels of employee performance by reinforcing employees' motivation, engagement, and willingness to contribute to organizational goals.

Employee performance is commonly defined as the outcomes achieved as a result of the effort invested in assigned tasks (Pradhan and Jena, 2017). Contemporary research conceptualizes employee performance as a multidimensional construct encompassing task performance, contextual performance, adaptive performance, and counterproductive work behavior (Koopmans et al., 2011; Pradhan and Jena, 2017; Hasinat et al., 2024).

Task performance refers to employees' proficiency in carrying out core job responsibilities and reflects the effectiveness with which individuals perform formally required activities (Campbell, 1993; Griffin et al., 2007). Contextual performance involves discretionary behaviors that support the social and psychological environment of the organization, such as helping colleagues, cooperating with team members, and facilitating collective functioning (Murphy, 1989; Campbell, 1993). Adaptive performance refers to employees' ability to adjust their behavior in response to changing work demands, including problem-solving, learning new skills, and adapting to new procedures or technologies (Chen et al., 2005; Park and Park, 2019). Counterproductive work behavior includes voluntary actions that may harm the organization or its members, such as reduced effort, withdrawal behaviors, or interpersonal deviance (Rotundo and Sackett, 2002).

Drawing on both the Social Exchange Theory and the AMO framework, employees who perceive HR practices as supportive and beneficial are more likely to reciprocate through positive work behaviors while reducing behaviors that may harm the organization. Accordingly, perceived HR practices are expected to influence the different dimensions of employee performance. Therefore, we posit that:

**H1**: Perceived HR practices have a positive effect on employees' task performance.

**H2**: Perceived HR practices have a positive effect on employees' contextual performance.

**H3**: Perceived HR practices have a positive effect on employees' adaptive performance.

**H4**: Perceived HR practices have a negative effect on employees' counterproductive work behavior.

### 2.2 Perceived human resource practices and affective commitment

Beyond their influence on behavioral outcomes such as employee performance, human resource practices also shape employees' psychological attachment to the organization. One of the most widely studied attitudinal outcomes in organizational research is affective commitment. Affective commitment refers to employees' emotional attachment to, identification with, and involvement in the organization (Camelo-Ordaz et al., 2011; Khandakar and Pangil, 2021). Employees with high levels of affective commitment tend to remain with the organization because they genuinely identify with its values and objectives.

From the perspective of Social Exchange Theory, the employment relationship can be understood as a reciprocal exchange process in which employees respond to favorable organizational actions with positive attitudes and behaviors. When employees perceive that the organization implements fair and supportive HR practices, they tend to reciprocate these investments by developing stronger emotional attachment and loyalty toward the organization. HR practices such as recruitment and selection, training opportunities, performance appraisal systems, reward and recognition mechanisms, and career development initiatives signal that the organization values employees' contributions and is committed to their professional growth. These signals foster perceptions of organizational support and fairness, which are key drivers of affective commitment (Alfes et al., 2012).

Empirical research provides substantial evidence supporting the positive relationship between HR practices and affective commitment. For example, Bashir and Venkatakrishnan (2022) found that several HR practices, including recruitment and selection, training, performance appraisal, rewards and recognition, and career development, were significantly associated with higher levels of affective commitment among employees working in micro, small and medium enterprises (SMEs) in India. Their results further indicated that career advancement opportunities represented one of the strongest predictors of employees' affective commitment.

Similarly, Hussien et al. (2022) reported that HR practices such as recruitment, training, socialization and job security significantly enhance employees' job satisfaction and affective commitment in the hospitality sector, highlighting the importance of effective HR systems in shaping positive employee attitudes. Cross-national evidence also supports this relationship. A comparative study conducted by Lee et al. (2018) demonstrated that perceived HR practices significantly predict affective commitment among employees in both the United States and South Korea. However, the relative importance of specific practices varies across contexts.

Taken together, these theoretical arguments and empirical findings suggest that when employees perceive HR practices as supportive, fair and development-oriented, they are more likely to develop a strong emotional attachment to their organization. Consequently, positive perceptions of HR practices are expected to enhance employees' affective commitment. Therefore, we posit that:

**H5**: Perceived HR practices have a positive effect on employees' affective commitment.

Affective commitment has also been identified as an important mechanism influencing employee behavior. Employees who feel emotionally attached to their organization are more likely to exert greater effort, engage in cooperative behaviors, and demonstrate higher levels of performance. At the same time, strong affective commitment tends to reduce the likelihood of negative behaviors, such as absenteeism and counterproductive work behaviors (Camelo-Ordaz et al., 2011; Luturlean et al., 2019).

Consequently, affective commitment may serve as a mediating mechanism linking perceived HR practices to employee performance. In other words, HR practices may enhance performance indirectly by strengthening employees' emotional attachment to the organization. Therefore, we posit that:

**H6**: Affective commitment partially mediates the relationship between perceived HR practices and task performance.

**H7**: Affective commitment partially mediates the relationship between perceived HR practices and contextual performance.

**H8**: Affective commitment partially mediates the relationship between perceived HR practices and adaptive performance.

**H9**: Affective commitment partially mediates the relationship between perceived HR practices and counterproductive work behavior.

### 2.3 Moderating role of transformational leadership

In organizational behavior research, leadership is widely recognized as a critical mechanism shaping employees' attitudes and behaviors at work. Among the various leadership styles examined in the literature, transformational leadership has received considerable scholarly attention because of its ability to inspire employees, strengthen intrinsic motivation, and align individual efforts with organizational objectives. Initially conceptualized by Burns (1978) and later extended by Bass (1985), transformational leadership refers to a leadership style in which leaders motivate followers to transcend their self-interest and commit to collective organizational goals through inspiration, intellectual stimulation, and individualized consideration.

A substantial body of empirical research demonstrates that transformational leadership exerts a positive influence on various dimensions of employee performance. Several studies have documented a direct relationship between transformational leadership and task performance. For instance, the empirical study conducted by Hussain et al. (2020) in the Pakistani telecommunications sector shows that transformational leadership has a significant positive effect on employee performance, as transformational leaders encourage employees to develop their competencies, share a common vision, and enhance the quality of their work. Similarly, research by Manzoor et al. (2019) within Pakistani SMEs indicates that transformational leadership is a significant predictor of employee performance because it strengthens employees' motivation and engagement.

Beyond formal task-related activities, the literature also highlights the capacity of transformational leadership to foster discretionary behaviors that contribute to organizational effectiveness, often conceptualized as contextual performance or organizational citizenship behavior. Empirical evidence provided by Qalati et al. (2022) demonstrates that transformational leadership significantly promotes organizational citizenship behaviors among employees in small and medium-sized enterprises. Their findings further reveal that such discretionary behaviors mediate the relationship between leadership and employee performance, suggesting that transformational leaders cultivate a cooperative and supportive environment in which employees voluntarily engage in behaviors that benefit the organization.

In addition to promoting positive behaviors, transformational leadership has been associated with a reduction in counterproductive work behaviors. Counterproductive behaviors, such as deliberate inefficiency, withdrawal, or actions that harm organizational interests, represent a significant obstacle to both individual and organizational performance (Karácsony and Czibula, 2020). Transformational leaders may act as a behavioral regulation mechanism by promoting ethical standards, reinforcing collective values, and inspiring employees to adopt constructive work attitudes.

Empirical evidence provided by Akbari et al. (2022) indicates that transformational leadership is negatively associated with counterproductive work behaviors, as employees working under transformational leaders are more likely to internalize organizational goals and display responsible work conduct. Similar conclusions were reported by Howladar et al. (2018), who found that transformational leadership mitigates the negative consequences of deviant workplace behavior by encouraging employees to focus on collective objectives. Comparable findings were also observed in public sector organizations by Baharom et al. (2017), who documented a significant negative relationship between transformational leadership and deviant workplace behaviors.

These empirical findings can also be theoretically interpreted through the lens of the Social Exchange Theory. According to this theoretical perspective, relationships within organizations are governed by reciprocal exchanges between employees and their leaders or organizations. When employees perceive that their leaders provide support, inspiration, and recognition, they feel a sense of obligation to reciprocate through positive attitudes and behaviors, including higher levels of performance and reduced engagement in counterproductive actions. Transformational leadership, therefore, functions as a key relational mechanism that activates reciprocal exchanges and encourages employees to respond with behaviors that benefit the organization.

Furthermore, prior research suggests that the influence of transformational leadership extends beyond routine job performance and encompasses more dynamic forms of performance, such as employees' ability to adapt to change and respond to evolving work demands. Transformational leaders stimulate employees intellectually, encourage them to challenge existing assumptions, and support them in developing new ideas and solutions. Through such mechanisms, transformational leadership contributes to enhancing employees' adaptive capacity and resilience in complex and uncertain work environments.

Taken together, the existing body of research consistently indicates that transformational leadership constitutes a critical driver of employee performance. By inspiring employees, fostering organizational commitment, and discouraging counterproductive behaviors, transformational leaders create a work environment conducive to high levels of individual performance. In this context, transformational leadership may also strengthen the effectiveness of human resource management practices. When employees perceive HR practices as supportive and fair, the presence of transformational leadership can reinforce these positive perceptions and amplify their impact on employees' performance-related behaviors.

Based on these theoretical arguments and empirical findings, it is reasonable to assume that transformational leadership can strengthen the relationship between perceived HR practices and the different dimensions of employee performance. Accordingly, the following hypotheses are proposed:

**H10**: Transformational leadership positively moderates the relationship between perceived HR practices and task performance, such that the relationship becomes stronger when transformational leadership is higher.

**H11**: Transformational leadership positively moderates the relationship between perceived HR practices and contextual performance, such that the relationship becomes stronger when transformational leadership is higher.

**H12**: Transformational leadership positively moderates the relationship between perceived HR practices and adaptive performance, such that the relationship becomes stronger when transformational leadership is higher.

**H13**: Transformational leadership strengthens the negative relationship between perceived HR practices and counterproductive work behavior, such that the negative effect becomes stronger when transformational leadership is higher.

**Figure 1:**
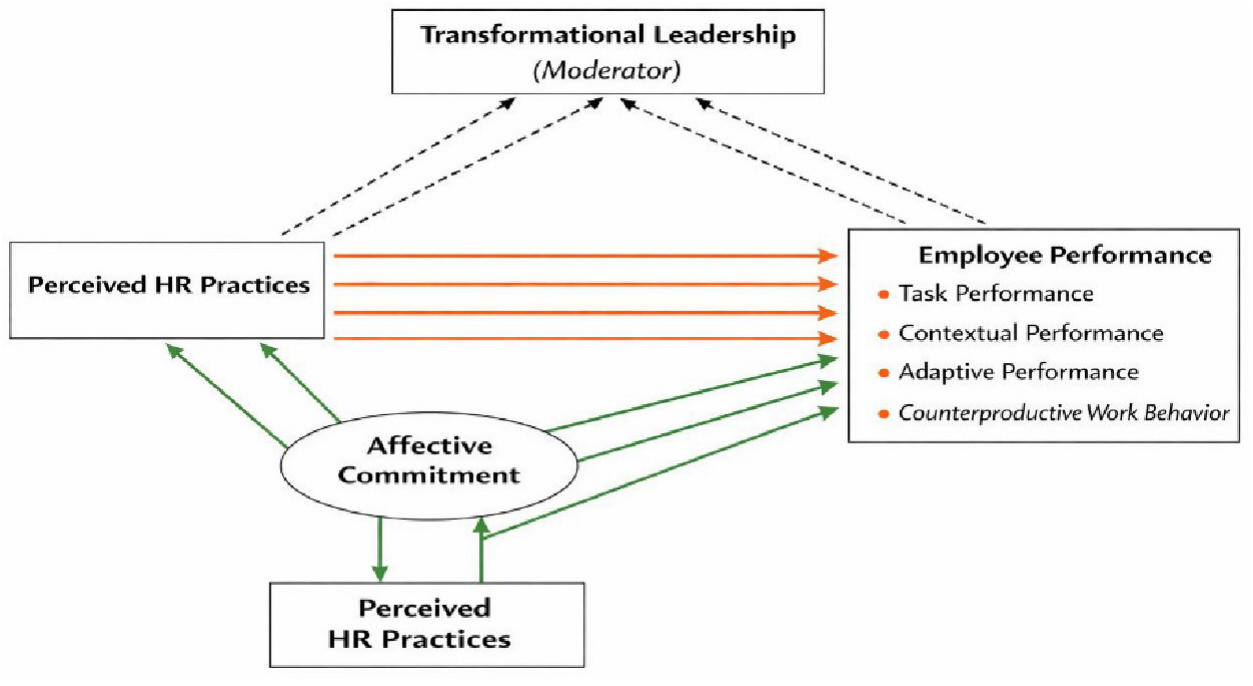
Conceptual research model.

Based on these theoretical arguments, the conceptual model presented in Figure 1 examines the direct effect of perceived HR practices on employee performance, the mediating role of affective commitment, and the moderating influence of transformational leadership.

## 3 Research design

This study adopts a quantitative, cross-sectional research design aimed at examining the relationships between perceived human resource management (HRM) practices, affective commitment, transformational leadership, and multidimensional individual performance. A cross-sectional design is considered appropriate when the objective is to test theoretically grounded relationships between latent constructs measured at a single point in time, particularly in organizational behavior research where perceptual variables are central (Hair et al., 2021; Saunders et al., 2019).

The study was conducted over a defined period, from early December 2025 to late February 2026, covering questionnaire development, data collection, and data analysis phases.

The empirical context is Morocco, an emerging economy characterized by institutional diversity and heterogeneous organizational structures. Emerging market contexts are particularly relevant for HRM research, as institutional environments shape the implementation and perception of HR practices, potentially leading to context-specific behavioral outcomes (Paauwe, 2004; Cooke et al., 2019; Boon et al., 2019). The sample includes employees working in both SMEs and large organizations, with a predominance of large firms, allowing for variation in organizational HR systems and practices.

A non-probability convenience sampling technique was employed due to the absence of a comprehensive sampling frame and the difficulty of accessing organizational employee lists. Although this approach limits statistical generalizability, it is widely accepted in HRM and organizational behavior research when studying perceptual constructs and dispersed populations, particularly in emerging economy contexts where access constraints are common (Etikan et al., 2016; Saunders et al., 2019). However, this sampling strategy implies potential selection bias, which is acknowledged as a limitation of the study.

Data were collected through an online questionnaire distributed via email and LinkedIn. This method was selected to ensure access to geographically dispersed employees and to facilitate participation across different organizational contexts. In addition, online data collection ensures anonymity and reduces social desirability bias, particularly when measuring sensitive perceptual constructs such as HRM practices, leadership behaviors, and employee attitudes. Nevertheless, online surveys may introduce self-selection bias and increase the risk of common method variance, as all variables are collected from a single source at a single point in time. These limitations are addressed through appropriate statistical controls in the data analysis phase.

A total of 152 valid responses were retained for analysis. While this sample size may appear limited, it is adequate for partial least squares structural equation modeling (PLS-SEM), which is particularly suitable for complex models, prediction-oriented research, and relatively small to medium sample sizes. PLS-SEM is widely recommended when the research objective focuses on theory development and model prediction rather than strict theory confirmation (Hair et al., 2021; Sarstedt et al., 2020).

## 4 Results

### 4.1 Respondent profile

Tables 1, 2 summarize the demographic and organizational characteristics of the sample, highlighting its overall heterogeneity. The respondents are slightly predominantly female (61%) and largely young, with 50% under 30 years old. Most hold graduate-level degrees (59%) and have relatively limited work experience, indicating a mainly early-career workforce.

**Table 1:** Demographic characteristics of respondents.

Variable	Category	Frequency	Percentage (%)
Gendre	Female	92	61%
	Male	60	39%
Age	< 30 years	76	50%
	30–40 years	63	41%
	>40 years	13	9%
Education level	Undergraduate (Bac+2 to Bac+3)	51	33%
	Graduate (Bac+4 to Bac+5)	89	59%
	Other	12	8%
Work experience	< 5 years	68	44%
	5–10 years	51	34%
	>10 years	33	22%

**Table 2:** Organizational characteristics of respondents.

Variable	Category	Frequency	Percentage (%)
Firm size	Large enterprise	96	63%
	Medium enterprise	37	24%
	Small and very small enterprises	19	12%
Sector of activity	Services (banking, telecom, distribution, tourism, and general services)	86	56%
	Industry (agri-food, mechanical, chemical, textile, and metallurgy, electronics)	49	32%
	Energy and construction	17	11%

From an organizational perspective, the sample is primarily composed of employees from large firms (63%) and is largely service-oriented (56%), followed by industry (32%). Overall, the sample is diverse yet structured, providing a relevant basis for interpreting the empirical results while reflecting specific contextual characteristics.

### 4.2 Measurement model assessment

Table 3 presents the reliability and convergent validity results of the measurement model. In accordance with the guidelines for PLS-SEM (Hair et al., 2021), the reliability and validity of the constructs were assessed using Cronbach's alpha, composite reliability (CR) and average variance extracted (AVE). All constructs demonstrate satisfactory internal consistency reliability, with Cronbach's alpha values above the recommended threshold of 0.70 and composite reliability values exceeding 0.80. Furthermore, the AVE values are above 0.50, confirming adequate convergent validity. These results indicate that the measurement model is reliable and suitable for structural model analysis.

**Table 3:** Measurement model: reliability and convergent validity.

Variables	Items	Cronbach's α	Composite reliability (CR)	AVE
Perceived training (Marwat et al., 2006)	1. I perceive that my organization provides extensive training programs that meet quality standards. 2. I perceive that those employees receive training programs regularly for their positions. 3. I perceive that training needs are identified through a formal performance evaluation process. 4. I perceive that formal training programs are provided to help new employees develop the skills required for their jobs. 5. I perceive that those employees regularly receive new skills and knowledge to improve teamwork. 6. I perceive that identified training needs are realistic and aligned with the organization's overall strategy.	0.84	0.88	0.57
Perceived compensation (Akbar and Rohmandiyas, 2021)	1. I perceive that the compensation I receive increases my motivation at work. 2. I perceive that the compensation I receive enhances my creativity at work. 3. I perceive that the compensation I receive improves my skills at work. 4. I perceive that the compensation I receive boosts my productivity at work. 5. I perceive that the compensation I receive improves the quality of my work.	0.93	0.95	0.80
Perceived recruitment (Gitonga and Njoroge, 2021)	1. I perceive that recruitment in my organization ensures the right person is placed in the right job. 2. I believe that clear information about the organization and the job is provided during recruitment. 3. I perceive that candidate selection is based primarily on merit. 4. I perceive that I was assigned to a position that matches my skills after recruitment. 5. I perceive that the organization provided adequate resources to support my work after recruitment. 6. I perceive that recruitment decisions prioritize employee competence. 7. I perceive that recruited employees have the necessary skills to perform effectively.	0.73	0.81	0.59
Perceived evaluation (Marwat et al., 2006)	1. I perceive that employee performance is measured using quantifiable results and objectives. 2. I perceive that those employees receive feedback and guidance to improve their performance. 3. I perceive that those employees trust the performance appraisal system. 4. I perceive that the performance appraisal system strongly influences individual and collective behavior. 5. I perceive that appraisal data are used to make decisions such as job rotation, training, and compensation.	0.79	0.86	0.54
Affective commitment (Meyer and Allen, 1991)	1. I would be very happy to spend the rest of my career with this organization. 2. I really feel as if this organization's problems are my own. 3. I do not feel ‘emotionally attached' to this organization. 4. This organization has a great deal of personal meaning for me. 5. I enjoy discussing my organization with people outside it. 6. I do not feel a strong sense of belonging to my organization. 7. I do not feel like “part of the family” at my organization.	0.88	0.91	0.60
Task performance (Koopmans et al., 2014)	1. I was able to plan my work so that it was completed on time. 2. I perceive that my work planning was optimal. 3. I kept in mind the results I needed to achieve in my work. 4. I was able to distinguish between primary and secondary problems at work. 5. I was able to perform my work effectively with minimal time and effort.	0.81	0.87	0.58
Contextual performance (Koopmans et al., 2014)	1. I took on additional responsibilities. 2. I started new tasks on my own when previous ones were completed. 3. I accepted challenging tasks when they were offered. 4. I worked to keep my professional knowledge up to date. 5. I continued to seek new challenges in my work. 6. I participated actively in work meetings.	0.86	0.89	0.56
Adaptive performance (Koopmans et al., 2012)	1. I demonstrated flexibility in my work. 2. I was able to manage difficult situations at work effectively. 3. I recovered quickly from challenging situations or setbacks at work. 4. I found creative solutions to problems. 5. I was able to cope well with uncertain and unpredictable situations at work. 6. I adapted easily to changes at work.	0.83	0.88	0.55
Counterproductive work behavior (Koopmans et al., 2014)	1. I complained about trivial matters at work. 2. I created bigger problems than they actually were at work. 3. I focused on the negative aspects of a work situation rather than the positive ones. 4. I talked with colleagues about the negative aspects of my work. 5. I talked with people outside the organization about the negative aspects of my work.	0.92	0.94	0.75
Transformational leadership (Singh and Krishnan, 2007)	1. My manager is open to criticism about himself/herself. 2. My manager encourages me to solve problems independently. 3. My manager tries to help me improve my weaknesses. 4. My manager gives credit and recognition to employees who deserve it or perform well. 5. My manager is attentive to my personal needs. 6. My manager shows great confidence in the abilities of his/her subordinates.	0.73	0.80	0.50

All values exceed the recommended thresholds (α ≥ 0.70; CR ≥ 0.70; AVE ≥ 0.50).

From a PLS-SEM perspective, the measurement model demonstrates satisfactory psychometric properties in line with established evaluation criteria. Cronbach's alpha values (≥0.73) and, more importantly, composite reliability coefficients (CR ≥ 0.80) indicate adequate to high internal consistency across all constructs. The AVE values (≥0.50) confirm convergent validity, suggesting that each latent variable explains a substantial proportion of the variance in its indicators. The constructs related to perceived HRM practices, as well as the different dimensions of individual performance, exhibit robust measurement properties, supporting the stability of the measurement model.

Similarly, affective commitment and transformational leadership meet the recommended thresholds, justifying their inclusion in the mediated and moderated structural model. Overall, the measurement model satisfies PLS-SEM methodological requirements and provides a reliable basis for estimating structural relationships.

### 4.3 Structural model assessment

#### 4.3.1 Direct effects

Following the assessment of the measurement model, the structural model was evaluated to test the proposed hypotheses. The significance of the structural relationships was assessed using a bootstrapping procedure with 5,000 resamples, following the recommendations of Hair et al. (2021). The results presented in Table 4 indicate that all hypothesized paths are statistically significant. Perceived HR practices have a strong positive effect on task performance (β = 0.573, *p* < 0.001), contextual performance (β = 0.575, *p* < 0.001) and adaptive performance (β = 0.575, *p* < 0.001). In contrast, perceived HR practices negatively influence counterproductive work behavior (β = −0.484, *p* < 0.001). In addition, perceived HR practices have a significant positive effect on affective commitment (β = 0.524, *p* < 0.001). These results support hypotheses H1–H5 and highlight the important role of perceived HR practices in shaping different dimensions of individual performance.

**Table 4:** Structural model results (direct effects).

Hypothesis	Relationship	B	*t*-value	*p*-value
H1	Perceived HR practices → task performance	0.573	>2.58	< 0.001
H2	Perceived HR practices → contextual performance	0.575	>2.58	< 0.001
H3	Perceived HR practices → adaptive performance	0.575	>2.58	< 0.001
H4	Perceived HR practices → counterproductive WB	−0.484	>2.58	< 0.001
H5	Perceived HR practices → affective commitment	0.524	>2.58	< 0.001

Standardized path coefficients are reported—significance assessed via bootstrapping (5,000 subsamples).

#### 4.3.2 Indirect effects

Table 5 presents the results of the mediation analysis examining the indirect effects of perceived HR practices on the different dimensions of individual performance through affective commitment. The results (Table 5) show that affective commitment significantly mediates the relationships between HR practices and all performance dimensions, with significant indirect effects (*t* > 3.90, *p* < 0.001). The presence of partial mediation indicates that HR practices affect employee performance both directly and indirectly through affective commitment, supporting its central explanatory role in the model.

**Table 5:** Mediation analysis (indirect effects).

Hypothesis	Mediated relationship	Indirect effect (β)	*t*-value	*p*-value	Type of mediation
H6	PHR → AC → task performance	0.29	4.18	< 0.001	Partial
H7	PHR → AC → contextual performance	0.33	5.02	< 0.001	Partial
H8	PHR → AC → adaptive performance	0.31	4.56	< 0.001	Partial
H9	PHR → AC → counterproductive WB	−0.27	3.94	< 0.001	Partial

Indirect effects tested using bootstrapping (5,000 resamples).

Table 6 reports the moderating effects of transformational leadership on the relationships between perceived HR practices and the different dimensions of individual performance. The moderation analysis (Table 6) shows that transformational leadership (TL) significantly strengthens the effects of HR practices on all performance dimensions, with significant interaction effects for task, contextual, and adaptive performance (*t* = 2.31–3.02, *p* < 0.05) and a significant negative interaction on counterproductive work behavior (*t* = 2.11, *p* = 0.035). These results confirm that TL enhances the positive impact of HR practices on desirable employee outcomes and attenuates counterproductive behaviors, highlighting its critical role as a boundary condition in the structural model.

**Table 6:** Moderating effects of transformational leadership.

Hypothesis	Moderated relationship	β (interaction)	*t*-value	*p*-value	Support
H10	PHR practices × TL → task performance	0.214	2.31	0.021	Yes
H11	PHR practices × TL → contextual performance	0.287	3.02	0.003	Yes
H12	PHR practices × TL → adaptive performance	0.259	2.68	0.007	Yes
H13	PHR Practices × TL → counterproductive work behavior	−0.182	2.11	0.035	Yes

Standardized path coefficients are reported. Significance was assessed using bootstrapping with 5,000 resamples.

#### 4.3.3 Interactions effects

**Figure 2:**
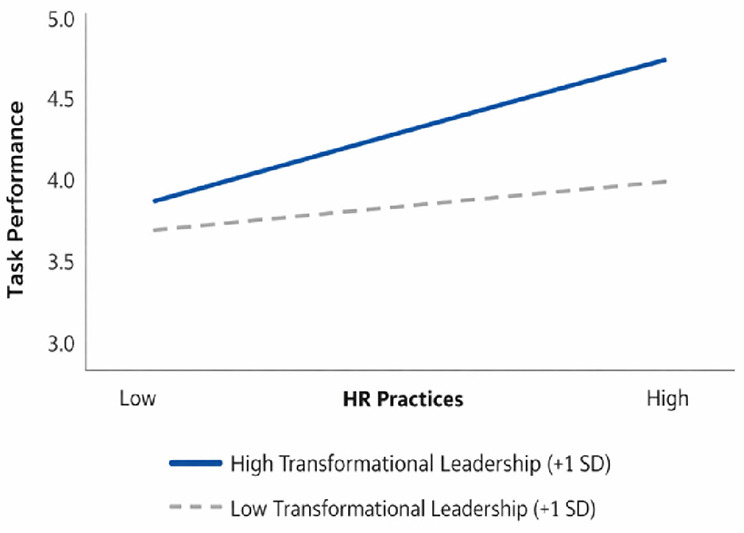
Moderating effect of transformational leadership on the relationship between HR practices and task performance.

**Figure 3:**
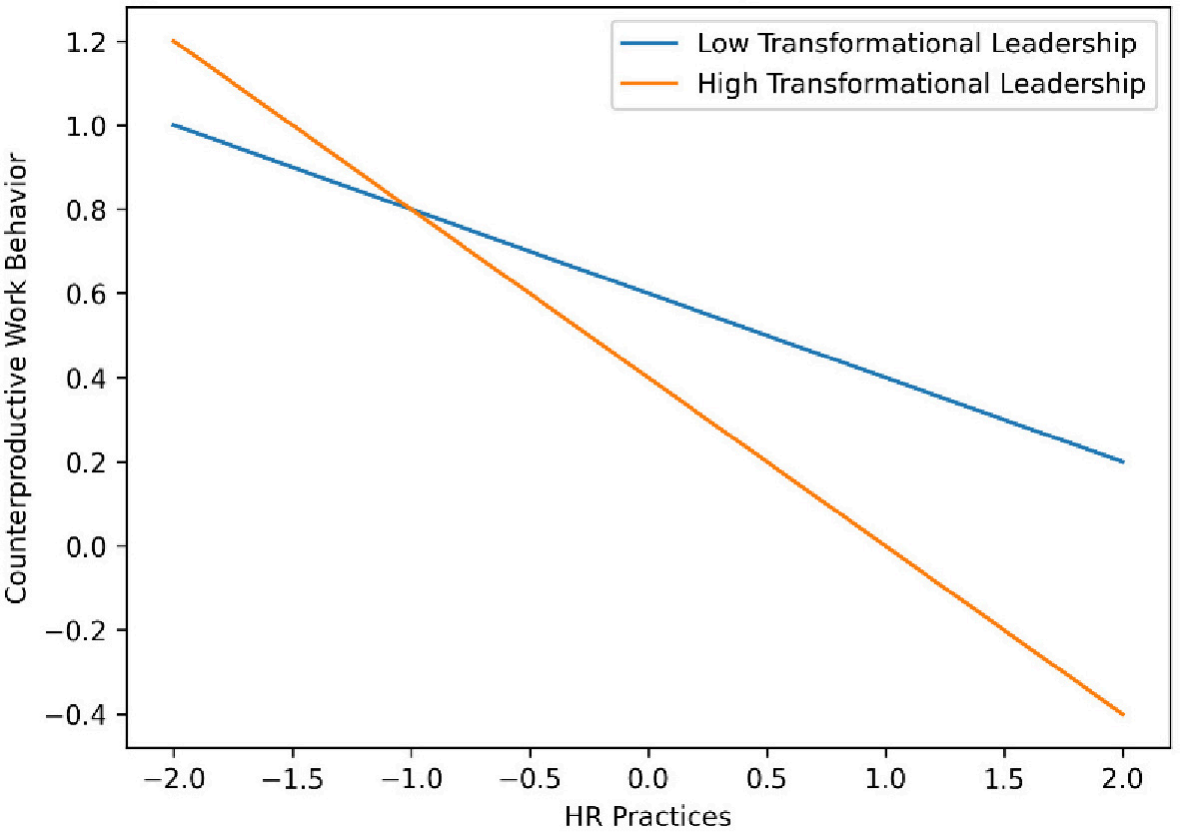
Moderating effect of transformational leadership on the relationship between HR practices and counterproductive work behavior.

To illustrate the moderation effects identified in the structural model, interaction plots are presented in Figures 2, 3. Figure 2 illustrates that the positive relationship between HR practices and task performance is stronger under high levels of transformational leadership than under low levels. To enhance clarity and avoid redundancy, only task performance is depicted as a representative outcome. The interaction patterns for contextual and adaptive performance are statistically similar and therefore not illustrated.

Figure 3 illustrates that the negative relationship between HR practices and CWB is stronger under high transformational leadership, indicating that effective HR practices are more effective in reducing CWB when supported by strong transformational leadership.

## 5 Discussion

This study examined how employees' perceptions of human resource management (HRM) practices influence multidimensional employee performance through affective commitment, and how this relationship is contingent upon transformational leadership. By integrating attributional, social exchange, and leadership perspectives, the study contributes to a more fine-grained understanding of the HRM–performance nexus in an emerging economy context.

A central finding is that perceived HRM practices are significantly associated with employee performance across its multiple dimensions, including task, contextual, adaptive performance, and counterproductive work behavior. This supports the growing consensus that performance cannot be adequately captured through a unidimensional lens, but rather reflects a constellation of in-role, extra-role, adaptive, and dysfunctional behaviors (Campbell, 1990; Motowidlo and Van Scotter, 1994; Rotundo and Sackett, 2002; Koopmans et al., 2011; Pradhan and Jena, 2017; Ramawickrama et al., 2017; Hasinat et al., 2024). The present findings further reinforce the argument that HRM systems shape not only task effectiveness but also broader behavioral patterns that are critical for organizational functioning in dynamic environments.

From a theoretical standpoint, these results support the HRM attribution perspective, which posits that employees do not respond to HR practices directly, but rather to the meanings they attribute to them (Nishii et al., 2008a; Wang et al., 2020). Consistent with social exchange theory (Blau, 1964; Cropanzano and Mitchell, 2005), favorable perceptions of HRM practices appear to generate reciprocal attitudinal and behavioral responses. However, the findings also extend this logic by demonstrating that HRM effects are not purely linear or automatic, but embedded in broader psychological and interpretive processes shaped by organizational context (Bowen and Ostroff, 2004; Paauwe, 2004; Purcell and Kinnie, 2007; Boxall and Macky, 2009; Den Hartog et al., 2013).

The study further confirms that affective commitment plays a significant partial mediating role in the HRM–performance relationship. This suggests that HRM systems operate through a dual pathway: a direct behavioral influence and an indirect attitudinal mechanism. Employees who perceive HRM practices as supportive, fair, and development-oriented are more likely to develop emotional attachment and identification with their organization (Meyer and Allen, 1991; Alfes et al., 2013; Lee et al., 2018; Khandakar and Pangil, 2021; Bashir and Venkatakrishnan, 2022). This finding aligns with broader HRM research emphasizing that employee attitudes represent a key mechanism through which HR systems translate into performance outcomes (Jiang et al., 2012; Guest, 2017).

Importantly, the partial mediation effect suggests the existence of additional intervening mechanisms beyond affective commitment. In particular, employee motivation and individual ability emerge as plausible complementary explanatory pathways, consistent with ability–motivation–opportunity (AMO) frameworks and broader HRM process models (Thabani, 2020; Mat Rani et al., 2021; Gazi et al., 2024). This highlights that HRM systems operate through a configurational rather than a singular causal logic, where multiple psychological states jointly translate HR practices into performance outcomes.

A further contribution of this study lies in identifying transformational leadership as a significant boundary condition. The results indicate that transformational leadership strengthens the positive relationship between perceived HRM practices and employee performance. This finding underscores the importance of leadership as an interpretive and sensegiving mechanism that shapes how HR practices are understood and enacted at the employee level (Burns, 1978; Bass, 1985; Crede et al., 2019; Hai et al., 2020). Transformational leaders, by fostering meaning, individualized consideration, and inspirational motivation, amplify the perceived credibility and salience of HRM practices, thereby enhancing their behavioral impact.

This moderating effect also reinforces the argument that HRM systems are not self-executing mechanisms. Rather, their effectiveness depends on complementary organizational processes, particularly leadership and communication systems that shape employee sensemaking (Bowen and Ostroff, 2004; Nishii et al., 2008a; Den Hartog et al., 2013). In this sense, the study contributes to opening the “black box” of HRM by demonstrating that HR–performance relationships are jointly shaped by perception-based mechanisms and contextual leadership conditions.

Overall, the findings suggest that HRM effectiveness emerges from a multi-level interplay between organizational practices, employee interpretations, and leadership dynamics. This supports a more nuanced view of HRM systems as socially constructed and contextually embedded rather than purely technical instruments of control or performance optimization (Paauwe, 2004; Boxall and Macky, 2009).

## 6 Future research directions

Future research could extend this study in several important directions. First, larger and more heterogeneous samples across sectors, organizational sizes, and employee categories would enhance external validity and improve the robustness of the model.

Second, mixed-method designs combining quantitative and qualitative approaches are recommended. While PLS-SEM captures structural relationships effectively, qualitative methods (e.g., interviews and case studies) would allow deeper exploration of how employees interpret HR practices and how such interpretations translate into behavior.

Third, comparative cross-country studies would be particularly valuable in order to assess the role of institutional and cultural environments in shaping HRM effectiveness, given that HR systems are highly context-dependent.

Finally, future research should integrate additional psychological mechanisms such as employee motivation, psychological empowerment, and ability-related constructs within a broader ability–motivation–opportunity (AMO) framework, in order to further unpack the “black box” of HRM–performance relationships.

## 7 Conclusion

This study examined the effect of employees' perceptions of HR practices on multidimensional employee performance, considering the mediating role of affective commitment and the moderating role of transformational leadership. Based on data collected from 152 employees and analyzed using PLS-SEM, the findings confirm the proposed theoretical model and highlight significant relationships among the studied variables.

The results demonstrate that employee performance cannot be fully explained by the implementation of HR practices alone. Rather, employees' perceptions, emotional attachment to the organization, and leadership context jointly shape behavioral and performance outcomes. Employees who perceive HR practices positively tend to develop stronger affective commitment, engage in positive work behaviors, and reduce counterproductive behaviors, while transformational leadership further amplifies these effects.

From a theoretical perspective, this study contributes to HRM literature by integrating perceived HR practices, affective commitment, and transformational leadership within a single moderated mediation framework. It also advances understanding of employee performance by adopting a multidimensional perspective.

From a managerial perspective, the findings suggest that organizations should not only design effective HR systems but also ensure their positive perception by employees and support their implementation through transformational leadership practices.

Despite its contributions, this study is subject to several limitations that should be carefully considered when interpreting the findings.

First, the study relies on a cross-sectional quantitative design based on employees' self-reported perceptions. While this approach is widely adopted in HRM research, it may introduce common method variance and limit causal inference. Although procedural remedies were applied and statistical checks suggest that common method bias is not a major concern, its possibility cannot be entirely ruled out. The reliance on a single source of data also raises concerns regarding perceptual bias. Future research could address these limitations by adopting longitudinal designs, multi-source data collection (e.g., supervisor-rated performance), or mixed-method approaches to enhance causal inference and reduce method-related bias.

Second, although the sample size (*n* = 152) is adequate for PLS-SEM and exploratory modeling, it remains relatively modest and may limit external validity. In addition, the sample is characterized by a predominance of young, highly educated employees working mainly in large, service-oriented organizations. This specific profile may influence how HR practices are perceived and enacted, thereby constraining generalizability, particularly to SMEs or industrial sectors. Future studies should replicate the model using larger and more heterogeneous samples across sectors, organizational sizes, and demographic groups.

Third, data were collected through an online questionnaire, which may introduce self-selection bias and reduce control over response conditions. Although this method enabled access to geographically dispersed respondents, it may also exclude certain employee categories. Future research could combine survey-based approaches with qualitative methods (e.g., interviews or case studies) and objective performance indicators to improve data robustness and triangulation.

Finally, this study does not incorporate other potentially relevant individual-level variables, such as employee motivation and ability, which are central components of ability–motivation–opportunity (AMO) frameworks. The exclusion of these variables may limit the explanatory power of the model. Future research should integrate these factors within more comprehensive configurational models. In addition, comparative cross-country studies would be particularly valuable to examine how institutional and cultural contexts shape the effectiveness of HR practices and leadership styles.

## Data Availability

The raw data supporting the conclusions of this article will be made available by the authors, without undue reservation.
